# Is burnout associated with work life conflict in medical resident?

**DOI:** 10.1192/j.eurpsy.2026.11522

**Published:** 2026-07-31

**Authors:** F. Dhouib, O. Errabia, N. Kotti, A. Kchaou, M. Hajjaji, K. Jmal Hammami

**Affiliations:** 1 Occupational medecine Department; 2 Family Medecine Department, Faculty of medecine of Sfax, Sfax, Tunisia

## Abstract

**Introduction:**

Burnout is a growing concern among medical residents, who are often exposed to long working hours, high clinical responsibilities, and limited personal time. These demanding conditions create significant work-life conflict, which may contribute to the development of burnout. Understanding the relationship between work-life conflict and burnout is essential for designing preventive strategies and promoting the well-being of future physicians.

**Objectives:**

This study aims to assess burnout in medical residents and highlight the association between burnout and work life conflict among this population.

**Methods:**

This was an analytical cross-sectional study conducted among Tunisian medical residents during the period from September 1, 2022 to December 31, 2022, through an online questionnaire. Work life interaction was assessed using the SWING (Survey Work-home Interaction NijmeGen) scale and burnout was assessed through Maslach Burnout Inventory. Statistical analysis were performed using the Statistical Package for the Social Sciences SPSS. The level of statistical significance was set at p<0.05.

**Results:**

During the study period, we interviewed 140 medical residents, with a mean age of 27.1±1.8 years with a predominance of female gender (67.8%). A proportion of 84.4% of the participants were single. Most of the participants in our study were first year residents (59.3%). In our sample, 32.9% of residents reported a high level of professional burnout. A similar proportion, 34.2%, indicated a high degree of depersonalization, as measured by the Maslach Burnout Inventory. Notably, 69.8% of residents reported a low level of personal accomplishment. According to the statistical analysis, a negative work life interaction and a negative life work interaction were associated with professional burnout and depersonalization, while a positive work life interaction and a positive life work interaction were correlated with personal accomplishment.

**Conclusions:**

Burnout is highly prevalent among medical residents, particularly in terms of diminished personal accomplishment. Strategies aimed at improving work–life balance and fostering positive interactions between professional and personal life may help reduce burnout and enhance residents’ well-being and performance.

**Disclosure of Interest:**

None Declared

